# Safety and Quality of Life in Women with Immediate Reconstruction with Polyurethane Implants after Neoadjuvant Chemotherapy: Outcomes from The Preq-20 Trial

**DOI:** 10.3390/cancers15041113

**Published:** 2023-02-09

**Authors:** Benigno Acea-Nebril, Alejandra García-Novoa, Carmen Cereijo-Garea, Carmen Conde Iglesias, Alberto Bouzón Alejandro, Carlota Díaz Carballada

**Affiliations:** 1 Breast Unit, Department of General Surgery, University Hospital Complex A Coruña, 15006 A Coruña, Spain; 2 Breast Unit, University Hospital Complex A Coruña, 15006 A Coruña, Spain; 3 Breast Unit, Ginecology Service, University Hospital Complex A Coruña, 15006 A Coruña, Spain

**Keywords:** prepectoral breast reconstruction, polyurethane implants, neoadjuvant chemotherapy

## Abstract

**Simple Summary:**

Neoadjuvant chemotherapy allows similar oncological control as its adjuvant administration in breast cancer. Many women undergoing neoadjuvant chemotherapy will require a mastectomy and there is little evidence of the safety of reconstruction in this group of patients. The aim of this study is to analyse the safety and satisfaction of women included in the PreQ-20 trial who underwent neoadjuvant chemotherapy and who were operated on with IPMR, compared with another patient group from the PreQ-20 trial who underwent an immediate postmastectomy reconstruction as primary surgery. The results of this prospective study will make it possible to determine the compatibility of this reconstruction with adjuvant treatments.

**Abstract:**

Introduction: Various studies have evaluated the impact of neoadjuvant chemotherapy (NAC) on the complications of breast cancer surgery, most of which were retrospective and did not assess the variables related to postoperative risk factors. The aim of this study is to analyse the safety and satisfaction of women included in the PreQ-20 trial who underwent NAC and who underwent mastectomy and immediate reconstruction with prepectoral polyurethane implants. Material and Methods: The patients included in the study belong to the prospective study PreQ-20. The study group consisted of patients who underwent immediate reconstruction after primary systemic therapy. The control groups consisted of patients with immediate reconstruction and adjuvant chemotherapy (control group 1) and patients with an infiltrating carcinoma or in situ ductal carcinoma who did not require chemotherapy (control group 2). Results: The study included 157 women, 58 (36.9%) of whom underwent primary systemic therapy. The indication for genetic study was significantly greater for the study group (87.9%) than for control groups 1 (49.1%) or 2 (30.4%). Seventy-two (45.9%) of the patients underwent bilateral mastectomy (BM), a procedure that was performed significantly more frequently in the study group (69%) than in control groups 1 (30.2%) or 2 (34.8%). The incidence rate for BM after complete pathologic response was 78%. There were no statistically significant differences in the number of complications between the groups. Implant loss was significantly more frequent in control group 1 (13.2%) than in the study group (3.4%) and control group 2 (2.2%). Conclusions: Mastectomy with prepectoral polyurethane implant reconstruction in patients with neoadjuvant chemotherapy presented a similar incidence of complications compared with patients who underwent primary surgery. There is a high rate of BM in women with NAC.

## 1. Introduction

NAC is a therapeutic modality that offers the same systemic control as its adjuvant administration to surgery [1,2,3]. Various clinical trials [1,2,3] have shown that the use of NAC provides opportunities for certain patients, such as systemic control for patients with locally advanced tumours, improvement in conditions for a conservative management of T3 tumours and for women with small breasts, and as a tumour sensitivity test to drugs, especially for those subtypes with a more aggressive biological component, such as triple negative and human epidermal growth factor receptor 2 (HER2) overexpression. However, more than 30% of patients will require a mastectomy after NAC and the vast majority of these women will undergo immediate reconstruction. Various meta-analyses [4,5,6] have evaluated the impact of NAC on the complications of breast cancer surgery. These meta-analyses have shown that NAC does not increase the risk of postoperative complications, although a number of authors have observed an increase in seroma [7] and implant loss [8,9]. These systematic reviews highlight the limited evidence in the analysed studies, given that a large portion of the studies are retrospective, with methodological heterogeneity, and most have not assessed variables related to postoperative risk factors, such as smoking, body mass index, mastectomy weight, prior radiation therapy and the delay between chemotherapy and surgery.

Immediate postmastectomy reconstruction (IPMR) is a valid and safe option for patients who undergo primary surgery after their diagnosis, but there is less evidence on its safety in women treated with NAC. Prepectoral breast reconstruction (PBR) has recently been proposed as a safe option in risk-reduction oncologic surgery due to its low morbidity and its safety in terms of postoperative complications and disease-free survival [10,11,12]. In this type of IPMR, the preservation of the pectoralis major muscle and the subcutaneous placement of the implant decreases postoperative pain and the incidence of postoperative complications, thereby constituting a good option for women with associated morbidity, as is the case for patients with NAC. However, we currently do not have an algorithm that allows choosing the appropriate method of reconstruction for each patient.

The aim of this study is to analyse the safety and satisfaction of women included in the PreQ-20 trial who underwent NAC and who were operated on with IPMR, compared with another patient group from the PreQ-20 trial who underwent an IPMR as primary surgery.

## 2. Patients and Method

The study included patients from the prospective study PreQ-20, whose objectives were to assess the safety, quality of life and cosmetic sequelae following an IPMR with polyurethane implants. The study has been assessed and approved by the Research Ethics Committee of A Coruña-Ferrol (code PreQ-20, reference number 2020/295). The study is registered in the ClinicalTrials.gov website (code NCT04642508) [13], and its protocol has recently been published [14].

### 2.1. Inclusion and Exclusion Criteria

The study included women diagnosed with breast carcinoma who underwent skin-sparing mastectomy (SSM) or nipple-sparing mastectomy (NSM) (unilateral or bilateral) and immediate prepectoral reconstruction with polyurethane implants. The study population included three patient groups:

- Study group: Patients diagnosed with infiltrating breast carcinoma who required systemic therapy with chemotherapy (with or without antibodies) prior to surgery. The indication for primary systemic therapy (PST) was agreed upon by the Breast Unit’s Tumour Committee. In the manuscript, the authors use NAC and PST as synonyms to define the patients in the study group who received chemotherapy (with or without antibodies) before surgery.
- Control group 1: Patients diagnosed with infiltrating breast carcinoma who underwent adjuvant systemic therapy with chemotherapy (with or without antibodies) after surgery.
- Control group 2: Patients diagnosed with an infiltrating carcinoma or in situ ductal carcinoma who did not require chemotherapy. In this group of women, the Tumour Committee assessed the indication for radiation therapy and adjuvant hormone therapy according to each patient’s staging and biological characteristics.

The study excluded women with neoadjuvant hormone therapy, disease progression during NAC and those patients who did not wish to participate in the study.

### 2.2. Preoperative Evaluation

All patients were assessed by the Breast Unit’s Tumour Committee for individual discussion of the indication for primary treatment, taking into account the staging of the process and the tumour’s biological characteristics. In this meeting, the committee selected the patients who, due to their family history or individual characteristics, were candidates for an assessment in the high-risk unit where the need for a genetic study was analysed. In addition to the mammogram and ultrasound, all study patients underwent magnetic resonance imaging (MRI) to confirm tumour size and dispersion, assess the distribution of glandular tissue and subcutaneous fat, as well as the subclavicular and sternal fat transitions. These studies facilitated the design of a mastectomy tailored to each patient’s anatomy. The patients in the study group underwent a second MRI after completing the chemotherapy to determine the degree of the disease’s clinical response.

### 2.3. Surgical Method

All patients underwent SSM or NSM, taking into account the criteria indicated by Nava et al. [15], according to which patients with small or medium breast volumes underwent NSM, except if the neoplastic process precluded NSM or if the suprasternal notch-to-nipple distance was greater than 25 cm. The patients with voluminous breasts underwent a Carlson type 4 SSM (Wise pattern) [16]. The mastectomy was performed according to the distribution of the neoplastic process in the patients with cancer and to the distribution of the subcutaneous fat shown in the preoperative MRI. The thickness of the skin flap was assessed according to the Rancati classification (breast tissue coverage classification) [17]: 1 cm for type 1, 2–3 cm for type 2 and ≥3 cm for type 3. This classification was used with the intention of predicting the cosmetic result after prepectoral reconstruction, in terms of visibility of the contour of the implant. The skin flap was sculpted according to each patient’s specific anatomy and the tumour’s location. Patients with NSSM underwent cleaning of the retroareolar tissue once the breast had been extirpated using the Folli technique [18]. The breast reconstruction was performed by placing a polyurethane foam-coated silicone implant (MicrothaneTM, POLYTECH Health & Aesthetics, Dieburg, Germany) in the prepectoral position. In all surgical procedures, antibiotic prophylaxis was administered with intravenous cefazolin during the first 24 h, and aspiration drainage was placed.

### 2.4. Postoperative Follow-Up

The patients were evaluated weekly for the first month in the Breast Unit office for early detection of complications. Subsequent follow-up was conducted every 6 months for the patients with cancer and annually for risk reduction. All patients underwent a breast MRI at 1 year of the surgery to assess the residual glandular tissue.

### 2.5. Neoadjuvant and Adjuvant Therapies

PST was indicated for those patients with locally advanced tumours (stage III), poor breast-to-tumour size ratio and tumour subtypes with a high probability of a full response to chemotherapy (triple negative, HER2 subtype). The chemotherapy consisted of a sequential regimen of 4 cycles of adriamycin and cyclophosphamide every 3 weeks followed by paclitaxel weekly for 12 cycles. Those patients with HER2 overexpression were treated with dual anti-HER2 blockade (pertuzumab and trastuzumab). Adjuvant chemotherapy was indicated according to the decision of our centre’s Tumour Committee using clinical guidelines. In most cases, the patients who required chemotherapy underwent a sequential regimen of 4 cycles of adriamycin and cyclophosphamide every 3 weeks followed by paclitaxel weekly for 12 cycles. Those patients with HER2 overexpression were prescribed trastuzumab to be taken every 3 weeks for 1 year. The patients with tumours that expressed hormone receptors underwent hormone therapy for 5 or 10 years, with tamoxifen for the premenopausal women and aromatase inhibitors for the postmenopausal women.

The indication for radiation therapy in the chest wall was performed by taking into account the final staging of the oncologic process and the proximity/involvement of the margin of the mastectomy. The radiation therapy dose was 45–50 Gy in 25 fractions of 1.8–2 Gy/day. For cases with affected margins without expansion, a radiation boost to the tumour bed at additional doses of 8–10 Gy, divided into 4 or 5 sessions, was indicated. Axillary radiation therapy was indicated for the women who had at least one of the following criteria: capsular rupture of the lymph node, lymph node involvement and absence of hormone receptor expression. The women who underwent axillary radiation therapy and/or in the supraclavicular region were administered a 50-Gy dose in 25 sessions, to a depth of 3 cm. The internal mammary chain was not irradiated in any case.

### 2.6. Evaluation of Satisfaction and Quality of Life

All study patients were given the preoperative module of the Breast-Q™ questionnaire (preoperative module of reconstruction) to determine their satisfaction and quality of life prior to the surgery. The members of the breast unit then gave the patients the postoperative module 12 to 24 months after the patients had completed the systemic therapy or radiation therapy for those patients with irradiation of the chest wall. The final score for each module was calculated according to the Mapi Research Trust criteria [19] and ranged from 0 to 100 (the higher the score, the greater the satisfaction).

### 2.7. Statistical Method

We performed a descriptive analysis of the variables included in the study. For the comparison of categorical variables, the Pearson test or the Fisher test was obtained, as appropriate. All quantitative variables are expressed as their mean and standard deviation. The qualitative variables are expressed as proportions. The means were compared using the Kruskal–Wallis test or ANOVA, as appropriate, after determining the normality with the Kolmogorov–Smirnov test. The survival curves were performed using Kaplan–Meier. The statistical analysis was performed using version 24 of the statistical program IBM SPSS and Epidat 4.1.

## 3. Results

The study included 157 women with breast cancer who underwent a mastectomy and immediate reconstruction with polyurethane implants in the prepectoral position from November 2018 to October 2022. The study group consisted of 58 women (36.9%) with PST; control group 1 consisted of 53 women (33.8%) who required adjuvant chemotherapy; and control group 2 consisted of 46 women (29.3%) who required no chemotherapy. The mean patient age was 47.2 years, and the study group was significantly younger than the other groups (Table 1). The indication for genetic study was significantly greater for the study group (87.9%) than for control groups 2 (30.4%) and 1 (49.1%) (*p* < 0.01). Twenty-two (37.9%) of the patients in the study group had a genetic mutation. NSM with inframammary fold incision was the most frequent procedure in all groups and was significantly more frequent in the study group (Table 2). The most common histological type in all groups was infiltrating ductal carcinoma, and the most common subtype was luminal B Her2- (Table 2). The time to the start of the postoperative therapies was significantly longer in control group 2.

### 3.1. Postoperative Complications

**Figure 1:**
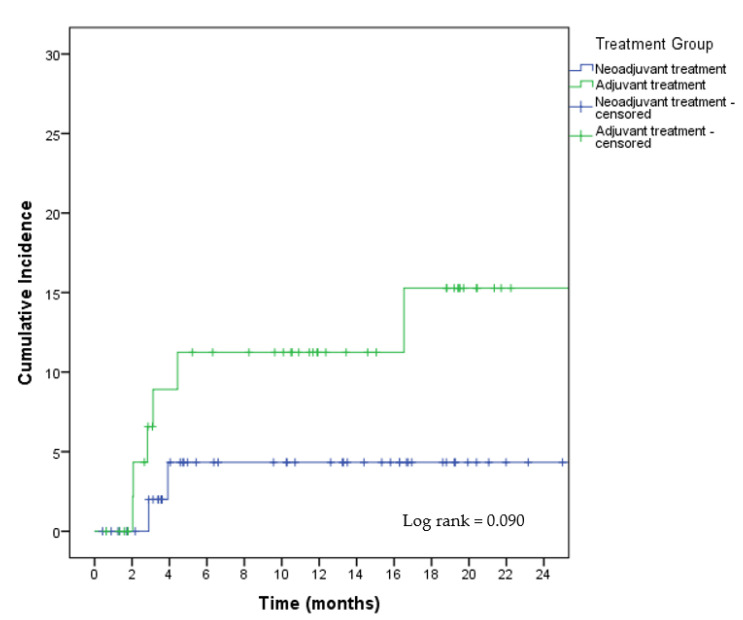
Actuarial incidence of implant loss.

There were no statistically significant intergroup differences in the number of complications (Table 3 ); however, there were more readmissions and reoperations in control group 1 (*p* = 0.014). In 10 (6.4%) women, the reconstruction failed due to implant loss, representing 4.4% of reconstructions. Implant loss was significantly more frequent in control group 1 (13.2%) than in the study group (3.4%) and control group 2 (2.2%) (*p* = 0.042). The actuarial incidence rate of implant loss at 12 months was 4.3% (95% CI 4–4.6%) in the study group and 11.2% (95% CI 6.5–15.9%) in control group 1 (log-rank, 0.09) (Figure 1).

### 3.2. Bilateral Mastectomy

Four women (2.5%) presented a bilateral breast carcinoma, with no intergroup differences (Table 1). Seventy-two (45.9%) of the patients underwent BM, a procedure performed significantly more frequently in the study group (69%) than in control groups 1 (30.2%) and 2 (34.8%; *p* < 0.01) (Table 2). Twenty-six (36.1%) of the patients with BM presented a genetic mutation: 13 in BRCA1, six in BRCA2, 2 in PALB2, two in ATM, one in p53, one in RAD51 and one in BRIP1. In the study group, the 22 patients with a genetic mutation (37.9%) and 18 women (31%) with mutation underwent BM. Ten of the patients without genetic mutation with BM had a complete radiological response. In control group 1, 16 women (30.2%) chose BM, three of whom presented a genetic mutation (2 in BRCA1 and 1 in BRCA2). In control group 2, 16 women (34.7%) underwent BM, one of whom presented a mutation in BRCA2 (Table 4).

The mean surgical time was 164.4 min and was significantly longer in the study group (*p* < 0.01). There were no significant intergroup differences for the surgical time of the BM (Table 2).

### 3.3. Oncologic Safety

The mean follow-up of the series was 14 months and was significantly shorter for the study group (10.9 months; *p* = 0.012) (Table 5). During the follow-up, three locoregional relapses were identified (1.9%), two of them (one in the axilla and the other in the mastectomy skin) in patients with triple-negative tumours and a complete response after PST. Four patients presented distant metastases (2.5%). Three of these patients belonged to the study group and were characterised as carriers of a genetic mutation, locally advanced tumours at diagnosis and a good response to systemic therapy (G4 and G5 of Miller and Payne). One of these patients died due to metastasis of their breast cancer (0.6%).

### 3.4. Satisfaction and Quality of Life

The patients in the study group showed significantly lower preoperative sexual satisfaction compared with the two control groups (Table 6). The total results of the Breast Q questionnaire showed no statistically significant differences between the preoperative assessment and that performed 1 year after completing the treatments (Table 7). However, the comparative analysis in each group showed a significant reduction in breast satisfaction in the women who underwent adjuvant therapy (*p* = 0.037). All of the patients included in this study presented significant worsening of their physical well-being 1 year after completing the treatments compared with the preoperative assessment (*p* = 0.003), although this was significant only in the patients with PST (*p* = 0.006).

## 4. Discussion

Mitosis inhibition is the main objective of chemotherapy, and its collateral damage is therefore focused on those physiological activities that depend on cell growth. Various clinical and experimental studies have reported these effects, showing reduced leukocyte production, inhibited fibroblast collagen production [20] and dysfunction of the vascular endothelium, promoting its adherence to platelets [21,22]. These effects are responsible for the increased susceptibility to infection, delayed healing and microcirculation thrombosis in breast cancer surgery after NAC. There is, therefore, controversy as to whether women with NAC are good candidates for IPMR, given that various studies have shown that this treatment, especially anthracyclines, causes an increase in postoperative complications due to the involvement of the immune system and damage to the vascular endothelium [20,21,22]. This limitation for immediate reconstruction represents a loss of opportunity for patients with NAC compared with deferred reconstruction for two reasons. The first is that deferred reconstruction does not include the preservation of anatomical elements of the original breast (skin coverage, inframammary fold, nipple–areolar complex), which optimises the cosmetic results in immediate reconstruction. The second reason is that deferred reconstruction in patients with NAC is performed on an irradiated chest wall, which requires the use of skin muscle flaps in most women. It is, therefore, especially important to assess the suitability of IPMR in patients who undergo NAC because the implementation of IPMR allows this group of women the possibility of SSM or NSM and breast reconstruction with implants, preferably in the prepectoral location.

However, the three meta-analyses that have evaluated the use of NAC prior to oncologic surgery [4] and to IPMR [5,6] have not identified cytostatics with a risk factor for postoperative complications or for disease-free survival and overall survival. The heterogeneity in the methodology and the retrospective analyses of many of the studies limit the level of evidence of these meta-analyses. The meta-analysis by Song [6] showed a low incidence rate of postoperative complications after NAC in young women, a patient group with a lower incidence of comorbidities. Our prospective study showed two noteworthy results related to postoperative complications. First, IPMR in patients with NAC presented a similar incidence of complications (13.8%) compared with the patients with adjuvant chemotherapy (16.9%) or without chemotherapy (10.9%). Second, the patients with adjuvant chemotherapy presented a significantly greater risk of readmission, reoperation and implant loss compared with the patients with NAC or without chemotherapy. The explanation for this fact is the increased incidence of wound dehiscence and necrosis of NAC in patients with adjuvant chemotherapy, which requires its surgical correction during therapy with cytostatics. Reoperation during the administration of chemotherapy results in implant infection and a lack of healing, a problem that does not occur in patients after NAC because their cytostatic therapy is completed 5–6 weeks earlier. Moreover, the infection rates in our study were low (1.9%) compared with most studies on reconstruction with prepectoral implants. Therefore, in the experiences with implants and biological meshes [23,24,25], infection and implant loss can affect 1.9–7.3% and 2.4–10.2% of the patients, respectively, while for synthetic meshes [26,27,28,29,30,31,32], the incidence of infection ranges from 0.8% to 6% and implant loss ranges from 1.2% to 8%. The explanation for this finding could be found in the use of the polyurethane implant in our study. We think that the low incidence of seroma and infection with these implants could be related to the integration of the polyurethane foam into the subcutaneous tissue, which allows implant adherence and tissue growth in its three-dimensional structure. This integration promotes resistance to infection in those cases of dehiscence or skin necrosis with exposure of the implant. This characteristic has enabled maintaining the reconstruction through the use of local flaps.

Another point of controversy is the choice of the most appropriate reconstruction technique for each patient. Various authors have described different methods of reconstruction: retropectoral with total muscle coverage, dual-plane retropectoral, prepectoral with mesh, prepectoral without mesh, among others [23,24,25,26,27,28,29,30,31,32]. The quality of the studies that have compared these reconstructive techniques is of low scientific evidence. However, most of the studies have demonstrated the lower morbidity of prepectoral reconstruction, its compatibility with oncological treatments and the good cosmetic result, with a natural appearance of the breast and without lively deformity. As Casella et al. [33] concluded in their retrospective study, there should be an algorithm that guides the surgeon in deciding the type of reconstruction that each patient should perform according to its clinical and morphological characteristics. In our opinion, prepectoral reconstruction seems to be a good alternative for most women.

A remarkable finding in our study was the high rate of BM in the patients with PST (69%), especially in those with complete response (78.2%). The increase in the indication for BM is a phenomenon already reported by various authors [34,35,36] over the last 20 years. These patients are characterised as women younger than 40 years, White, non-Hispanic and with private health insurance. Furthermore, the increase in BM after PST has been shown, in a study by Pollon et al. [37], in a review of 59,568 patients with immediate reconstruction after NAC between 2010 and 2014. Although the incidence rate of complete pathological responses increased from 33% to 46% during this period, conservative surgery only increased from 37% to 40%, while BM with reconstruction increased from 8% to 13%. The explanation for this finding could be related to the genetic studies performed during the PST, which in our experience 88% of patients underwent, providing most of these patients with information to help with decision-making prior to surgery. Thus, 36% of the patients with PST presented a mutation, identifying them as women at high risk for developing breast and ovarian cancer. Additionally, the fact of having been tested for a genetic mutation can increase awareness of the risk of new breast tumours and serve as a factor favouring BM in these patients’ decision-making. However, these arguments related to the assessment and awareness of the genetic risk do not explain the paradox that the incidence of BM in patients with G5 and G4 Miller and Payne responses after PST were 78.2% and 72.2%, respectively. We believe that, in this patient group, there are additional factors that affect this decision. On one hand, preoperative chemotherapy represents an arduous experience that most women do not wish to repeat and, unlike patients with adjuvant chemotherapy, have the opportunity to choose BM to reduce the likelihood of a second chemotherapy. On the other hand, there are generational implications that affect the decision-making, given that many of these patients belong to the millennial generation (1982–2004), characterised by a more active role in shared decision-making, affinity for proven and up-to-date information, and a preference for consulting videos for their information [38]. Additionally, these patients actively participate in social networks in which prophylactic contralateral mastectomy is a trending topic with intense debate, as shown in the study by Mamor et al. [39]. In this study, 155,000 Facebook users (mostly young women) published, shared and reshared 163,200 articles related to prophylactic contralateral mastectomy between April and May 2017. In our clinical experience, these patients have knowledge of the genetic risks and the benefits of prepectoral reconstruction, given that they have seen them on YouTube. In this area, it is important to highlight that the information obtained by patients in digital media may lack scientific evidence. In a study carried out by Marcasciano et al. [40] that analysed the content of different web pages, it was evidenced that their content lacks precise information on the risks of prepectoral reconstruction and its possible complications. For this reason, it is essential that health professionals contrast the information obtained by patients and guide them in decision-making. In our opinion, it is important to consider these generational implications, given that these women will enter screening programmes for breast cancer in the next 5–10 years and will possibly propose radical options for early tumours. Additionally, it is predicted that this generation will experience an increased incidence of breast cancer (and especially colon cancer) due to the fact that their diet during childhood was rich in fat and carbohydrates [41].

Our unit has previously evaluated the quality of life of patients with conservative surgery [42], oncoplastic surgery [43,44] and immediate reconstruction [45]. The present study shows three noteworthy results reported by the patients. First, the preoperative sexual well-being was significantly changed in the patients with PST compared with the patients with primary surgery, possibly influenced by the toxic effects of chemotherapy. Second, there were no changes in breast satisfaction before and after the surgery in the women with PST, unlike the patients with adjuvant chemotherapy, whose satisfaction decreased after surgery. Our study identified this worsening by evaluating the satisfaction before and after surgery in each patient, unlike most studies that only evaluated postoperative satisfaction and therefore lacked an initial reference. Third, there was a worsening of physical well-being at 1 year of the surgery, especially among the patients with PST, which is related to deterioration caused by the drugs (chemotherapy, hormone therapy, antibodies) compared with the start of the process. None of the previously mentioned meta-analyses evaluated the quality of life and satisfaction of patients who underwent reconstruction after NAC, although most of the meta-analyses [12,46,47] agree that prepectoral reconstruction provides better results in terms of satisfaction and quality of life than retropectoral reconstruction.

This study has several limitations. The follow-up time was short and did not allow for an adequate assessment of the patients’ overall survival and disease-free survival. The postoperative quality-of-life assessment had fewer questionnaires due to the fact that many of the study patients had not yet reached one year of follow-up after the surgery.

## 5. Conclusions

In conclusion, IPMR with polyurethane implants in patients with NAC presented a similar incidence of complications compared with the patients with primary surgery. The readmission, reoperation and implant loss rates were lower for the patients with PST (5.2%, 3.4%, 3.4%, respectively) compared with those who underwent adjuvant chemotherapy (20.8%, 18.9%, 13.2%, respectively). There is a high rate of BM among the women with PST (69%), even in the presence of a complete pathologic response (78%), because the majority (88%) have undergone a genetic study during PST and a number of them (36%) presented a mutation. Lastly, the satisfaction and quality of life of the patients with IPMR after PST had not changed at 1 year of follow-up, although there was worsening of the physical well-being, as in the other evaluated groups.

## Figures and Tables

**Table 1:** Patients’ clinical characteristics.

Patient Characteristics	Study Group *n* = 58	Control Group 1 *n* = 53	Control Group 2 *n* = 46	*p*	TOTAL *n* = 157
Age (years)	43.3 (±7.6) (30–66)	48.3 (±8.7) (29–69)	50.9 (±9.3) (36–79)	<0.01 *	47.2 (±9) (29–79)
Menstrual state					
Premenopausal	49 (84.5%)	39 (73.6%)	34 (73.9%)	NS **	122 (77.7%)
Postmenopausal	9 (15.5%)	13 (26.4%)	12 (26.1%)		34 (33.3%)
Laterality					
Right breast	29 (50%)	22 (41.5%)	23 (50%)	NS **	74 (47.1%)
Left breast	28 (48.3%)	29 (54.7%)	22 (47.8%)		79 (50.3%)
Bilateral	1 (1.7%)	2 (3.8%)	1 (2.2%)		4 (2.5%)
Metachronic					
Tumour bed	0 (0.0%)	2 (3.8%)	3 (6.5%)	0.154 **	5 (3.2%)
Unscarred breast	1 (1.7%)	4 (7.5%)	6 (13.0%)		11 (7.0%)
Contralateral breast	2 (3.4%)	1 (1.9%)	1 (2.2%)		4 (2.5%)
Genetic study					
Not requested	7 (12.1%)	27 (50.9%)	32 (69.6%)		66 (42.0%)
Pending	5 (8.6%)	16 (30.2%)	5 (10.9%)		26 (16.6%)
BRCA1	11 (19.0%)	2 (3.8%)	0 (0.0%)		13 (8.3%)
BRCA2	4 (6.9%)	1 (1.9%)	1 (2.2%)		6 (3.8%)
Li–Fraumeni syndrome	1 (1.7%)	0 (0.0%)	0 (0.0%)		1 (0.6%)
PALB2	2 (3.4%)	1 (1.9%)	1 (2.2%)		4 (2.5%)
RAD51	1 (1.7%)	0 (0.0%)	0 (0.0%)		1 (0.6%)
BRIP1	1 (1.7%)	0 (0.0%)	0 (0.0%)		1 (0.6%
ATM	2 (3.4%)	0 (0.0%)	0 (0.0%)		2 (1.3%)
Negative study	24 (41.4%)	6 (11.4%)	7 (15.2%)	<0.01 **	37 (23.5%)
BMI, kg/m^2^	24.3 (±4.8)	24.6 (±5.1)	23.2 (±3.8)	NS *	24.2 (±4.7)
Sternum–NAC distance	19.0 (±3.2)	19.2 (±3.4)	19.9 (±2.7)	NS *	20.2 (±3.4)

Abbreviations: BRCA, breast cancer gene; BMI, body mass index; NAC, nipple–areolar complex. * Kruskal–Wallis test; ** X^2^ Pearson test.

**Table 2:** Patients’ surgical characteristics.

Surgical Features	Study Group	Control Group 1	Control Group 2	*p*	TOTAL
	*n* = 58	*n* = 53	*n* = 46		*n* = 157
Bilateral Mastectomy	40 (69.0%)	16 (30.2%)	16 (34.8%)	<0.01 **	72 (45.9%)
Type of mastectomy					
Stewart lower pole	0 (0.0%)	1 (1.9%)	0 (0.0%)	0.026 **	1 (0.6%)
SSM I	7 (12.1%)	5 (9.4%)	2 (4.3%)		14 (8.9%)
SSM II	0 (0.0%)	1 (1.9%)	0 (0.0%)		1 (0.6%)
SSM IV	12 (20.7%)	5 (9.4%)	4 (8.7%)		21 (13.4%)
NSM inframammary	34 (58.6%)	24 (45.3%)	23 (50%)		81 (51.6%)
NSM vertical	5 (8.6%)	17 (32.1%)	17 (36.9%)		39 (24.8%)
Axillary surgery					
No study	1 (1.7%)	3 (5.7%)	5 (10.9%)	<0.01 **	9 (5.7%)
SLNB	43 (74.1%)	27 (50.9%)	41 (89.1%)		111 (70.7%)
Lymphadenectomy	14 (24.1%)	23 (43.4%)	0 (0.0%)		39 (24.8%)
Oncologic breast weight (g)	308.4 (±201.0)	349.4 (±281.3)	243.5 (±183.8)	0.055 *	301.7 (±228.9)
Contralateral breast weight (g)	326.6 (±220.9)	278.6 (±307.0)	249.1 (±263.8)	0.206 *	297.9 (±252.9)
Surgical time (min)	182.5 (±53.7)	169.8 (±47.1)	139.0 (±48.0)	<0.01 *	164.4 (±52.7)
Unilateral m.	148.1 (±32.1)	157.9 (±44.2)	123.5 (±31.3)	0.02 *	143.1 (±40.2)
Bilateral m.	196.3 (±54.9)	194.3 (±44.2)	168.1 (±60.3)	0.213*	189.1 (±54.6)
Mean stay	1.9 ± 0.8	1.5 ± 0.6	1.6 ± 0.5	0.30 *	1.7 ± 0.7
Histological type					
DCIS	0 (0.0%)	0 (0.0%)	14 (30.4%)	<0.01 **	14 (8.9%)
IDC	56 (96.6%)	41 (77.4%)	24 (52.2%)		121 (77.1%)
ILC	2 (3.4%)	10 (18.9%)	7 (15.2%)		19 (12.1%)
Other	0 (0.0%)	2 (3.8%)	1 (2.2%)		3 (1.9%)
Tumour subtype					
Luminal A	0 (0.0%)	16 (30.2%)	15 (32.6%)	<0.01 **	31 (19.7%)
Luminal B Her2-	22 (37.9%)	22 (41.5%)	15 (32.6%)		59 (37.6%)
Luminal B Her2+	12 (20.7%)	8 (15.1%)	0 (0.0%)		20 (12.7%)
Her2+	2 (3.4%)	3 (5.7%)	0 (0.0%)		5 (3.2%)
Triple Negative	22 (37.9%)	4 (7.5%)	0 (0.0%)		26 (16.6%)
Not valid	0 (0.0%)	0 (0.0%)	16 (34.8%)		16 (10.2%)
Tumour size (pT)					
Tis	0 (0.0%)	0 (0.0%)	14 (30.4%)	<0.01 **	0 (0.0%)
T1mic	0 (0.0%)	0 (0.0%)	2 (4.3%)		2 (1.3%)
T1a	10 (17.2%)	2 (3.8%)	1 (2.2%)		13 (8.3%)
T1b	5 (8.6%)	9 (17%)	4 (8.7%)		18 (11.5%)
T1c	9 (15.5%)	14 (26.4%)	14 (30.4%)		37 (23.6%)
T2	8 (13.8%)	20 (37.7%)	10 (21.7%)		38 (24.2%)
T3	1 (1.7%)	8 (15.1%)	1 (2.2%)		10 (6.4%)
Axillary staging (pN)					
Nx	1 (1.7%)	3 (5.7%)	5 (10.9%)	<0.01 **	9 (5.7%)
N0	41 (70.7%)	17 (32.1%)	36 (78.2%)		94 (59.9%)
N1mic	1 (1.7%)	6 (11.3%)	4 (8.7%)		11 (7.0%)
N1	10 (17.2%)	17 (32.1%)	1 (2.1%)		28 (17.8%)
N2	3 (5.2%)	8 (15.1%)	0 (0.0%)		11 (7.0%)
N3	2 (3.4%)	2 (3.8%)	0 (0.0%)		4 (2.5%)
Time from the surgery to the next treatment (days)	49.8 ± 115.1	42 ± 17.8	66.0 ± 14.6	0.004 *	47.1 ± 18.4
Radiation therapy	36 (62.1%)	33 (62.3%)	13 (28.3%)	<0.01 **	82 (52.2%)

Abbreviations: SSM, skin-sparing mastectomy; NSM, nipple-sparing mastectomy; SLNB, sentinel lymph node biopsy; DCIS, ductal carcinoma in situ; IDC, invasive ductal carcinoma; ILC, invasive lobular carcinoma. * Kruskal–Wallis test; ** X^2^ Pearson test.

**Table 3:** Postoperative complications.

Complication Type	Study Group *n* = 58	Control Group 1 *n* = 53	Control Group 2 *n* = 46	*p*	Total *n* = 157
Postoperative complications	8 (13.8%)	9 (16.9%)	5 (10.9%)	0.681 **	22 (14.0%)
Haematoma	2 (3.4%)	1 (1.9%)	2 (4.3%)		4 (2.5%)
Abscess	3 (5.2%)	0 (0.0%)	0 (0.0%)		3 (1.9%)
Breast seroma	2 (3.4%)	2 (3.8%)	1 (2.2%)		5 (3.2%)
Wound dehiscence	0 (0.0%)	4 (7.5%)	0 (0.0%)		4 (2.5%)
Partial necrosis of the NAC	0 (0.0%)	1 (1.9%)	0 (0.0%)		1 (0.6%)
Skin necrosis	2 (3.4%)	2 (3.8%)	1 (2.2%)		5 (3.2%)
Rash	0 (0.0%)	0 (0.0%)	1 (2.2%)		1 (0.6%)
Readmission	3 (5.2%)	11 (20.8%)	3 (6.5%)	0.014 **	17 (5.7%)
Reoperation	2 (3.4%)	10 (18.9%)	3 (6.5%)	0.014 **	15 (3.8%)
Implant Loss	2 (3.4%)	7 (13.2%)	1 (2.2%)	0.042 **	10 (6.4%)

Abbreviations: NAC, nipple–areolar complex. ** X^2^ Pearson test.

**Table 4:** Type of mastectomy according to the genetic study and treatment type.

Unilateral or Bilateral Mastectomy	Study Group	Control Group 1	Control Group 2	*p*	Total
	*n* = 58	*n* = 53	*n* = 46		*n* = 157
Bilateral mastectomy					
Genetic mutation					
Yes	22 (37.9%)	3 (5.7%)	1 (2.2%)	<0.01 **	26 (16.6%)
No	18 (31.0%)	13 (24.5%)	15 (32.6%)		46 (29.3%)
TOTAL	40 (69.0%)	16 (30.2%)	16 (34.8%)		72 (45.9%)
Unilateral mastectomy					
Genetic mutation					
Yes	0 (0.0%)	1 (1.9%)	1 (2.2%)	0.749 **	2 (1.3%)
No	18 (31.0%)	36 (67.9%)	29 (63.0%)		83 (52.9%)
TOTAL	18 (31.0%)	37 (69.8%)	30 (65.2%)	30 (65.2%)	85 (54.1%)

** X^2^ Pearson test.

**Table 5:** Events during follow-up.

Events	Study Group *n* = 46	Control Group 2 *n* = 58	Control Group 3 *n* = 53	NO Systemic Therapy *n* = 46	*p*	Total *n* = 157
Mean follow-up (months)	16.8 ± 10.8	10.9 ± 10.2	15.1 ± 11.5	16.8 ± 10.8	0.012 *	14.0 ± 11.0
Locoregional relapses	1 (2.2%)	2 (3.4%)	0 (0.0%)	1 (2.2%)	-	3 (1.9%)
Distant metastasis	0 (0.0%)	3 (5.2%)	1 (1.9%)	0 (0.0%)	-	4 (2.5%)
Deaths	0 (0.0%)	1 (1.7%)	0 (0.0%)	0 (0.0%)	-	1 (0.6%)

* Kruskal–Wallis test.

**Table 6:** Preoperative Breast Q questionnaire (30 patients who did not answer the survey were excluded).

Preoperative Satisfaction	Study Group *n* = 49	Control Group 1 *n* = 41	Control Group 2 *n* = 37	*p*	Total *n* = 127
Satisfaction with the breast	66.7 ± 20.9	66.9 ± 22.8	68.9 ± 23.1	0.911 *	67.4 ± 22.0
Psychosocial well-being	68.7 ± 19.6	70.4 ± 18.2	73.5 ± 19.3	0.571 *	70.7 ± 19.0
Physical well-being	74.4 ± 14.6	68.7 ± 14.4	72.8 ± 12.3	0.116 **	72.1 ± 14.0
Abdomen assessment	77.8 ± 15.8	80.3 ± 21.2	83.1 ± 17.0	0.406 *	80.1 ± 18.0
Sexual satisfaction	56.1 ± 20.7	61.1 ± 21.9	71.0 ± 22.3	0.007 *	62.1 ± 22.3

* Kruskal–Wallis test; ** ANOVA.

**Table 7:** Postoperative Breast Q questionnaire.

Postoperative Satisfaction	Study Group *n* = 16	Control Group 1 *n* = 9	Control Group 2 *n* = 14	*p*	Total *n* = 39
Satisfaction with the breast	66.4 ± 19.6	60.3 ± 13.6	71.5 ± 19.2	0.369*	66.9 ± 18.3
Outcome	83.8 ± 15.3	74,0 ± 21.1	81.6 ± 16.8	0.398 *	80.7 ± 17.3
Psychosocial well-being	79.9 ± 20.5	68.4 ± 18.8	76.6 ± 16.1	0.398 *	76.1 ± 18.7
Physical well-being	64.9 ± 19.9	68.0 ± 9.7	69.9 ± 11.5	0.672 *	67.5 ± 14.9
Abdomen assessment	79.5 ± 28.9	79.3 ± 20.5	84.3 ± 17.6	0.959 *	81.6 ± 18.2
Sexual satisfaction	63.3 ± 24.6	53.3 ± 13.6	66.3 ± 18.1	0.473 *	63.1 ± 20.2
Nipple	97.3 ± 5.5	50	100	0.112 *	93.4 ± 17.5
Information	86.1 ± 17.1	77.7 ± 20.2	81.9 ± 16,2	0.518 *	82.7 ± 17.4
Surgeon	94.4 ± 15.4	98.3 ± 5.0	91.0 ± 17.3	0.221 *	94.2 ± 14.3
Medical team	96.9 ± 5.9	94.6 ± 16.3	100	0.165 *	97.4 ± 8.7
Office equipment	96.3 ± 12.8	95.3 ± 14.0	100	0.436 *	97.3 ± 10.6

* Kruskal–Wallis test.

## Data Availability

Data not available due to ethical constraints.
